# 3D Bioprinting for Spinal Cord Injury Repair

**DOI:** 10.3389/fbioe.2022.847344

**Published:** 2022-04-20

**Authors:** Tian-Yang Yuan, Jun Zhang, Tong Yu, Jiu-Ping Wu, Qin-Yi Liu

**Affiliations:** Department of Spine Surgery, The Second Hospital of Jilin University, Jilin University, Changchun, China

**Keywords:** 3D bioprinting, scaffolds, spinal cord injury repair, neural system tissue engineering, hydrogels

## Abstract

Spinal cord injury (SCI) is considered to be one of the most challenging central nervous system injuries. The poor regeneration of nerve cells and the formation of scar tissue after injury make it difficult to recover the function of the nervous system. With the development of tissue engineering, three-dimensional (3D) bioprinting has attracted extensive attention because it can accurately print complex structures. At the same time, the technology of blending and printing cells and related cytokines has gradually been matured. Using this technology, complex biological scaffolds with accurate cell localization can be manufactured. Therefore, this technology has a certain potential in the repair of the nervous system, especially the spinal cord. So far, this review focuses on the progress of tissue engineering of the spinal cord, landmark 3D bioprinting methods, and landmark 3D bioprinting applications of the spinal cord in recent years.

## 1 Introduction

Spinal cord injury (SCI) is considered to be one of the most challenging central nervous system injuries, the serious complications and high rates of paraplegia caused by SCIs have brought great burden to individuals, families, and society (Venugopal et al., 2017). Once damaged or degenerated, nerve cells cannot be self-repaired, which results in the permanent loss of function. From a pathological point of view, SCI is caused by a primary injury and a series of secondary injuries. Primary injuries are mainly acute injuries caused by mechanical forces such as extrusion and dislocation, including damage to neurons and glial cells at corresponding segments, leading to the rupture of blood vessels (Schwab and Bartholdi, 1996; Sobrido-Camean and Barreiro-Iglesias, 2018). Secondary injuries are relatively complicated including ion homeostasis disorders, local edema, ischemia, free radical excess, and intense inflammatory responses (Rowland et al., 2008; Orr and Gensel, 2017). At the same time, both primary injuries and secondary injuries to the microenvironment are not conducive to the differentiation of neurons (Sabelstrom et al., 2014). This series of changes can lead to the formation of cystic cavities, while astrocyte proliferation leads to the formation of scar tissue (Liu and Chan, 2016). In the presence of cavities, the formation of glial scars further impedes the regeneration of neurons, eventually leading to the loss of original sensation and voluntary activity.

At present, the clinical treatment of spinal cord injury is mainly divided into non-surgical treatment and surgical treatment. One of the non-surgical treatments for SCIs is post-injury shock therapy with excessive doses of methylprednisolone (MP) (Breslin and Agrawal, 2012), which is a corticosteroid that inhibits lipid peroxidation. While acting as a scavenging agent for free radicals, it also restricts the inflammatory response, protecting the spinal cord–blood barrier, and increasing blood flow to the damaged spinal cord. However, since its therapeutic benefits are also controversial—including increasing risks of urinary tract, respiratory, and wound infections—these complications limit its use (Hurlbert, 2014). For surgical treatment, decompression and fixation have always been the most important methods to treat SCIs. The primary goal of SCIs treatment is to remove the compression factors of and restore the stability of spinal structure to the greatest extent. Multisystem medical management, hypothermia, and rehabilitation medical care are also the methods of treating SCIs (Silva et al., 2014). The aforementioned methods have made some progress in the repair of SCIs; however, at present, all clinical treatment methods can only remove the injury factors to the greatest extent but cannot make the injured nerve regenerate functionally. Therefore, it can be said that its repair of the injury is incomplete.

Nervous system regeneration refers to the re-establishment and repair of functional neural connections, nervous tissue, and cells. Methods to accomplish this through neural tissue engineering involve providing direct replacement of neural cells and/or the repair of circuitry by utilizing cell transplantation, bio/chemical molecular signaling, and directed guidance “bridge” scaffolding (Joung et al., 2020). The most fundamental part is the regeneration of cells. There are only three major cell types in the spinal cord, and all cell lineages can be derived from neural stem cells (NSCs). These three kinds of cells are neurons, astrocytes, and oligodendrocytes. The axon extends out of the neuron and forms a network with other neurons. After being electrically stimulated, the axon terminals release neurotransmitters to form chemical signals and transmits the chemical signals to the dendrites of other neurons to which they are functionally connected. These functional connections are called synapses, which form the basis of the neuronal network (Joung et al., 2020). Glial cells-astrocytes or oligodendrocytes play a supporting role in the structure of the spinal cord. Oligodendrocytes mainly form myelin around neurons, which plays a vital role in the electrochemical activities of neurons. Among the many phenotypes of astrocytes, scar-forming astrocytes mainly form glial scar which produce axonal growth inhibitors and prevents axonal regeneration. This sequential phenotypic change has long been considered to be unidirectional and irreversible; thus glial scarring is one of the main causes of the limited regenerative capability of the SCIs (Okada et al., 2018). Consequently, many researchers have focused on the directed regeneration of neurons in the repair of SCIs, hoping to reduce the formation of glial scar as much as possible while regenerating neurons, and expect the regenerated neurons to form synapses with the host neurons, so as to finally restore the function of the damaged site.

Researchers began by injecting NSCs and/or cell growth factors directly into the site of the injury, but the study did not consider introducing scaffolds into the injury site. Although these studies achieved good results in animal experiments, the lack of scaffolding structure and difficulties in the selection of optimal performance cells have been the great challenges to translate this method into practical clinical application (Joung et al., 2020). With the development of tissue engineering, researchers consider that the repair of SCIs needs to provide a certain support structure to the damaged site, to restore the mechanical structure, and provide an effective protective barrier for implanted cells (Struzyna et al., 2014; Struzyna et al., 2015).

## 2 The “Requirements” for Spinal Cord Injury Scaffold

The spinal cord is linear in structure, which provides structural support for the body’s reflex response, the axons extending from neurons form synapses with other neurons, which is the basis of body’s reflex response. For example, the corticospinal tract is the most important motor system of human beings, but the corticospinal projection remains largely refractory to regeneration. The fundamental cause of this failed regeneration is that synapses cannot be formed between neurons (Kadoya et al., 2016).

For the repair of SCIs, the ideal biomimetic scaffolds should satisfy the following criteria:

1) The designed biological scaffold should conform to the mechanical properties of the spinal cord as far as possible, which is ideally approximately 10 kPa (Joung et al., 2020).
2) It should be conducive to cell adhesion, proliferation, and differentiation.
3) It should be biodegradable.
4) It should have an appropriate channel diameter because channels larger than ≈450 µm in diameter resulted in decrease in nerve regeneration (Joung et al., 2020).
5) Most importantly, it should have compatibility.

In order to achieve such ideal conditions, the most important things are scaffold fabrication materials and the scaffold fabrication process. First, soft materials are needed to make the scaffolds to avoid mechanical mismatch. Hydrogels are widely used in the application of scaffolds because most of them have good biocompatibility and predictable biodegradability (Huang et al., 2017), and most natural hydrogels have specific cell binding sites, which are required for cell attachment, proliferation, growth, and differentiation (Gungor-Ozkerim et al., 2018). Hydrogels can also facilitate the exchange of gases and soluble nutrients (Yeh et al., 2006). At the same time, hydrogel is more suitable for the strength of spinal scaffolds because of its low mechanical properties, and because of their high water content and non-toxicity, excellent mimics of the extracellular matrix (ECM) is rendered (Holzl et al., 2016).

They can be broadly divided into two categories—that is, natural and synthetic hydrogels. Natural hydrogels—such as collagen, chitosan, agarose, and alginate—have been widely used because of their good biocompatibility. They can be further classified based on their source. Hydrogels such as collagen, fibrin, and gelatin typically come from vertebrates, and therefore have inherent cell-adhesion signaling molecules, while hydrogels such as alginate and agarose come from other organisms, such as algae, which lack these signaling molecules (Hospodiuk et al., 2017). For example, collagen contains some adhesion motifs as RGD (Arg–Gly–Asp) -an important tripeptide for the interaction between a variety of cells and the ECM (Gasperini et al., 2014). Agarose does not have adhesion motifs to cells, so when it acts as a hydrogel matrix, there is no interaction with cells (Tang et al., 2007). However, it can also be supplemented cell adhesion motifs to the matrix, such as fibronectin (Karoubi et al., 2009) or RGD soluble peptide (Guaccio et al., 2008). Sharp et al. (2012) treated a rat model of SCI with salmon fibrin, the results demonstrated that the sensory, motor, and bladder function of the rats recovered to a certain extent after treatment. Ansorena et al. (2013) made progress in an experiment using alginate to conduct nerve growth factor repair in the spinal cord hemi-section model. In their experiment, the hydrogel scaffold-carrying growth factor had a certain effect on the functional recovery of animals after SCIs.

Although natural hydrogels have good biocompatibility, their very soft mechanical properties cannot provide the strength of biological tissues, which can easily cause structural collapse after implantation, leading to repair failure. Conversely, synthetic hydrogels can be chemically modified with domains that enhance the mechanical properties of biological printing structures. For example, adding methacryloyl groups into gelatin to form a synthetic hydrogel–gelatin methacrylate (GELMA), the hydrogel can exhibit different mechanical strengths under different ultraviolet light times and intensities. At the same time, the mechanical strength can also be adjusted according to the concentration of methacryloyl groups (Gungor-Ozkerim et al., 2018). The degradability of the synthetic hydrogels can also be modulated. For example, McKinnon et al. (2013) synthesized a polypeptide cross-linked poly (ethylene glycol) (PEG) hydrogel and investigated the proliferation and differentiation of embryonic stem cell-derived motor neurons (ESMNs) in it. Their result demonstrated that the hydrogels were able to promote neuronal survival and axon outgrowth using cell-extracellular matrix interactions and allowed neurons to remodel their extracellular environment using matrix metalloproteinase (MMP)-mediated hydrogel degradation. In addition, improved synthetic hydrogels can carry a variety of bioactive factors that can affect the proliferation and differentiation of NSCs. For example, research works printed fibroblast growth factor-2 (FGF2), ciliary neurotrophic factor (CNTF), and fetal bovine serum (FBS) on a polyacrylamide-based hydrogel. After NSCs were seeded on the scaffold, NSCs were proven to respond properly to printed macromolecules, and suggested that printed scaffold could successfully achieve effective control of stem cell differentiation (Ilkhanizadeh et al., 2007).

Another factor that has an impact on scaffolds fabrication is the manufacturing process. The research on tissue engineering scaffolds is not uncommon. The traditional manufacturing methods of tissue engineering scaffolds, such as gas foaming, melt molding, electrospinning, and phase separation, have been used in the production of scaffolds composed of synthetic and natural polymers (Liao et al., 2004; Cheng et al., 2018).

Bioprinting technology has attracted extensive attention in recent years. Bioprinting is defined as the use of 3D printing technology to deposit bioink or biomaterials ink on a receiving solid or gel substrate or liquid reservoir (Demirci and Montesano, 2007; Mironov et al., 2008; Moon et al., 2010).3D printing technology is an emerging technology, which can accurately “reproduce” tissue using the computer-aided design (CAD). 3D printing provides four important features (Joung et al., 2020) as follows:

1) It can be combined with 3D imaging technology to achieve anatomical accuracy.
2) Its robot-based bio-manufacturing methodology helps to achieve printing precision.
3) It is compatible with a variety of material groups to achieve flexible functionality.
4) It affords rapid prototyping to achieve combinatorial sampling.

It is different from traditional “subtractive” manufacturing, which processes the raw material, removing some of it to shape the tissue as required. “Additive” manufacturing, on the other hand, uses layers of materials that are combined to create a target tissue sample. In recent years, it has rapidly developed in the field of biomedical science and has been effectively applied in areas such as the osteochondral interface, peripheral nerves, kidneys, skin, cardiovascular, livers, and artificial ears (Mannoor et al., 2013; Johnson et al., 2015; Kesti et al., 2015; Homan et al., 2016; Gungor-Ozkerim et al., 2018; Rastogi and Kandasubramanian, 2019).

With the development of 3D printing, many researchers have focused on the theoretically feasible combination of SCI repairs using this technique. First, the combination of 3D printing and 3D images can customize the shape of the scaffold based on the distinctive SCI of different individuals, forming a “personalized” manufacturing scheme, so that the printed tissue structure can fit the microenvironment of the injury and achieve maximum anatomical fidelity. At the same time, 3D printing can provide direction for the regeneration of axons. This directional regeneration is conducive to the formation of synapses between axons, so as to achieve the regeneration of neural pathways in the spinal cord, and then promote the formation of neural networks (Kadoya et al., 2016). Second, 3D printing technology can be combined with multi-nozzle technology to achieve the function of printing multiple materials on a single printer at the same time. The combination of these different materials allows the structural simulation at the injury site to more closely resemble the natural spinal cord structure (Cao et al., 2003; Mironov et al., 2003; Hollister, 2005; Fedorovich et al., 2007; Ladd et al., 2013; Tabatabai et al., 2013). Furthermore, bioprinting has advantages in the establishment of *in vitro* models, as compared with traditional tissue engineering, bioprinting samples can be formed faster (Johnson et al., 2016). The establishment of *in vitro* models brings “preoperative simulation” to the reconstruction of nervous system tissue more effectively, which can achieve the goals of injury repair more accurately and effectively. Consequently, compared with traditional tissue engineering, 3D printing technology has created the potential for personalized tissue manufacturing, offering a new therapeutic trend for SCI regeneration.

The concepts of bioink and biomaterials ink are similar. The main difference is that the former, where cells belong to the mandatory component of the printing formulation, they can also contain bioactive factors. The latter, biomaterials are used for printing, and the cell contact occurs after manufacturing (Groll et al., 2018). Therefore, bioprinting can produce bioactive scaffolds with cell seeding by combining 3D printing with biomaterials ink; “living scaffold” or “cell-laden” can also be produced by combining 3D printing with bioink (Joung et al., 2020). Due to the limitations of the traditional scaffold fabrication process, which are not mild, it is impossible to make scaffolds containing bioactive factors, let alone the living scaffold. Even if the cells are cultured after scaffold fabrication, the growth direction of cells cannot be effectively controlled. At the same time, the scaffolds made by the traditional “subtraction” process cannot have a specific shape, such as 3D grid, which is composed of multi-dimensional linear structure. Most of the scaffolds manufactured by them only have porous structure on the surface of the scaffolds, while the interior is mostly solid structure. As such, traditional tissue engineering can be challenging in its quest to mimic the complexity of the spinal cord (Faroni et al., 2015).

In this article, we summarize the application of bioprinting in SCI repairs and show how this technique has led to further advances in neural tissue engineering. First, we introduce several main methods for spinal cord bioprinting. Second, we present landmark research on spinal cord bioprinting in recent years. Finally, we discuss future application prospects and the related disadvantages of this technique.

## 3 Bioprinting Methods for the Nervous System

**FIGURE 1:**
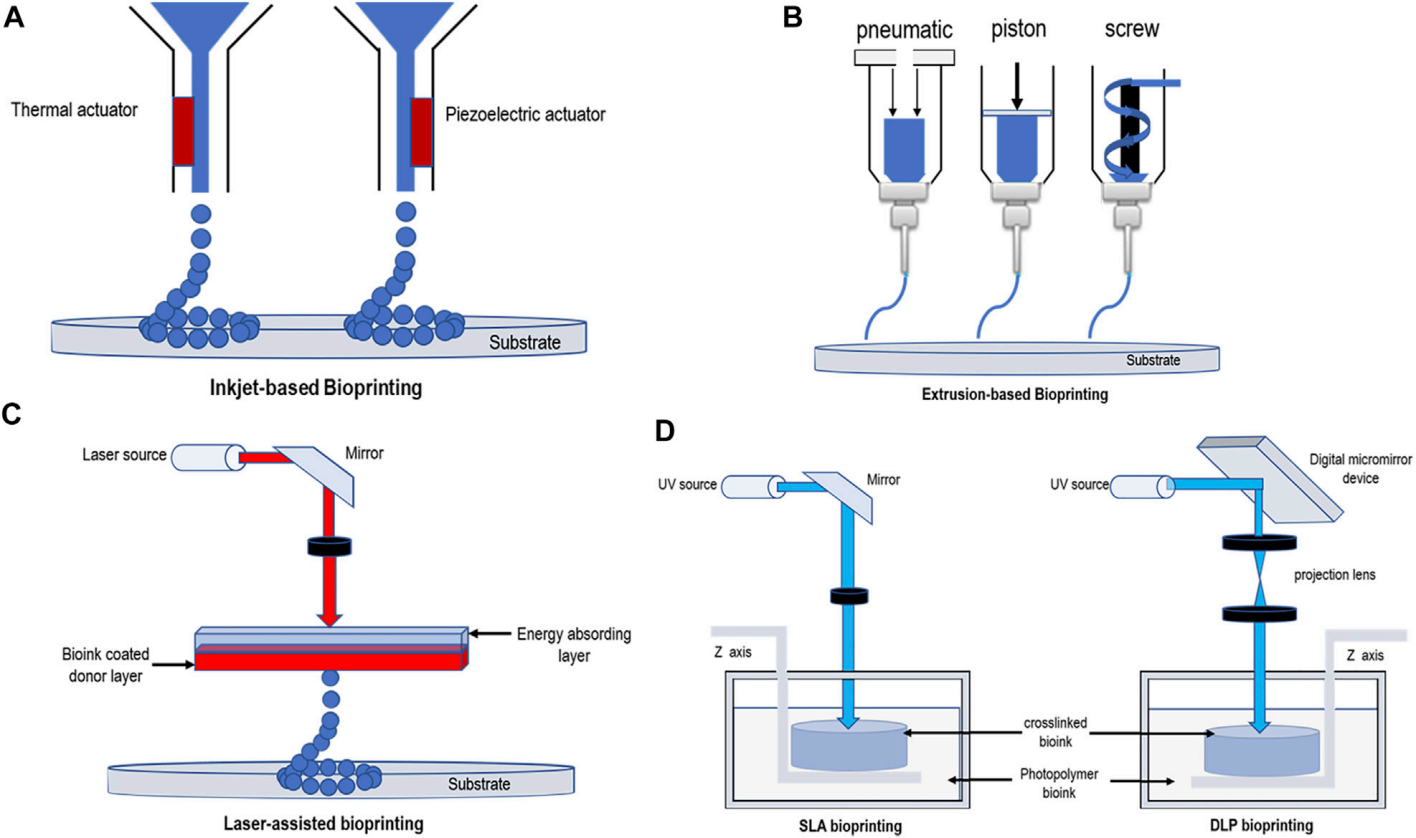
Schematic diagrams demonstrating bioprinting methods. **(A)** Thermal and piezoelectric inkjet-based bioprinting. **(B)** Extrusion-based bioprinting (pneumatic pressure, piston, and screw). **(C)** Laser-assisted bioprinting. **(D)** SLA bioprinting and DLP bioprinting.

Bioprinting of the nervous system can be broadly divided into the following categories—that is, inkjet/droplet bioprinting, extrusion bioprinting, and light-assisted bioprinting (Khalil and Sun, 2009; Wust et al., 2011; Dababneh and Ozbolat, 2014; Wang et al., 2015). These methods are illustrated schematically in Figure 1, and the comparison of different bioprinters is listed in Table 1.

**TABLE 1:** Comparison of different bioprinter.

	Inkjet bioprinting	Extrusion-based bioprinting	Laser-assisted bioprinting	DLP	SLA
Print process	Drop-by-drop	Line-by-line	Dot-by-dot	Continuous	Continuous
Print speed	Fast (Murphy and Atala (2014); Zhu et al. (2016); Bishop et al. (2017)	Slow (Murphy and Atala (2014); Zhu et al. (2016); Bishop et al. (2017)	Medium (Murphy and Atala (2014); Zhu et al. (2016); Bishop et al. (2017)	Fast (Zhu et al. (2016))	Fast but slow preparing speed (Bishop et al. (2017); Daly et al. (2021))
Resolution	20–100 μm (Chang et al. (2011))	100 μm (Ozbolat and Yu (2013); Derakhshanfar et al. (2018))	10 μm (Derakhshanfar et al. (2018))	1 μm (Kim et al. (2018))	50–100 μm (Gauvin et al. (2012); Gou et al. (2014); Wang et al. (2015))
Cell viability	＞85% (Xu et al., 2005; Xu et al. (2006))	＞90% (Seol et al. (2014); Liu et al. (2021))	＜85% (Hopp et al. (2012))	85–95% (Zhu et al. (2016))	＞90% (Gauvin et al. (2012); Gou et al. (2014))
Viscosity	<10 MPa/s air bubbles (Calvert, 2001; Holzl et al. (2016))	30 MPa/s to 6 × 107 MPa/s (Chang et al. (2011); Holzl et al. (2016))	1–300 MPa/s (Murphy and Atala (2014); Jungst et al. (2016))	No limitation	No limitation
Advantages	High print speed and high resolution	Ability to print high cell densities models	Does not produce shear force at the nozzle-head, good cell viability	No artificial interfaces and no limitation on cell viscosity; high resolution	No limit on the cell viscosity value; possible to create highly complex geometrical features
Disadvantages	Low cell viscosity and density; relatively low cell viability	Slow print speed; relatively low cell viability	Long print times and low cell viability	The hydrogel suitable for this technology remains to be explored	Bioink must be photo-cross-linkable; damage to cells during photo curing

### 3.1 Inkjet Bioprinting

Inkjet bioprinting, which permits the simultaneous and contactless deposition of cells in certain directions at micrometer resolutions (Zheng et al., 2011), can be divided into thermal, piezoelectric actuator, laser-induced forward transfer, and pneumatic pressure methods (Nahmias et al., 2005; Boland et al., 2006; Lee et al., 2009). Thermal and piezoelectric methods are the principal methods used for inkjet bioprinting (Seol et al., 2014). In thermal inkjet printers, the temperature provides heat to the print nozzle which in turn creates a pulse of air pressure that allows droplets to be ejected. Several studies have demonstrated that this localized heating,which can range from 200 to 300°C, does not have a substantial impact on the stability of biological molecules or on the viability or post-printing function of mammalians (Xu et al., 2005; Xu et al., 2006). The printing speed of this method is rapid, and a tissue size of between 20 and 100 μm can be printed (Chang et al., 2011). Piezoelectric inkjet printers use piezoelectric actuators, the speed and direction of droplet generation being determined by controlling the timing, pulse frequency, and amplitude of the actuators (Murphy and Atala, 2014). Compared with thermal printing, piezoelectric printing causes less damage to cells, resulting in a higher cell survival rate as there is no heat source (Cui et al., 2010). Moreover, there are researchers who have examined the use of acoustic inkjet printers, which use an acoustic radiation force associated with an ultrasound field to eject liquid droplets from an air–liquid interface (Demirci and Montesano, 2007; Fang et al., 2012). This printing method can generate and control droplets of uniform size and direction, avoiding exposure to pressure and thermal stress. At the same time, acoustic inkjet printers can combine multiple ejectors to print a variety of cell and material types (Nakamura et al., 2005; Saunders et al., 2008; Tasoglu and Demirci, 2013).

A common drawback of inkjet bioprinting is that because the tissue produced using this printing method does not have high viscosity, the technique can only dispense bioinks with <10 MPa/s air bubbles (Calvert, 2001; Holzl et al., 2016), the cell viscosity being maintained between 3.5 and 12 MPa/s (Chang et al., 2011; Murphy and Atala, 2014), which often results in poor mechanical properties, making it difficult to maintain the shape to withstand external stresses after implantation (O'Brien et al., 2015; Seol et al., 2014). Another limitation encountered by the users of inkjet-based bioprinting technology is that small nozzle dimensions and flow rates limit the volume accumulated per drop (<10 pL). This means that high cell concentrations (>5 million cells/mL) must be seeded to maximize the probability that each bioink drop will contain a cell (O'Brien et al., 2015), that is, cell density that can be printed using bioprinting technology is low, usually < 10^6^ cells/mL (Mandrycky et al., 2016). Moreover, recent studies have shown that the settling effect—that is, when bioink starts in the cartridge it can be well mixed, but during the printing process the cells begin to precipitate in the cartridge, thereby increasing the ink density, resulting in potential nozzle blockages (Pepper et al., 2011; Pepper et al., 2012; Mandrycky et al., 2016). This technology has a certain challenge for spinal cord bioprinting at present because the inkjet method cannot produce structures with complex accuracy, and because of the discontinuous mode of drop-by-drop, it is also difficult to manufacture cell-laden scaffolds or living scaffolds.

### 3.2 Extrusion-Based Bioprinting

Among all the existing 3D bioprinting techniques, extrusion-based printing has been the most widely adopted technique (Liu et al., 2017; Shen et al., 2020). Using this technique, a mixture of cells and hydrogels is printed using a micro-nozzle, allowing the material to be “extruded” into the substrate. The micro-extruder uses CAD–CAM instructions to stack the material onto a substrate in the form of beads. First, the beads are placed in the X-Y direction, after which the extruder head is moved along the Z-axis to create a complex 3D structure (Peltola et al., 2008; Murphy and Atala, 2014). Consequently, compared to inkjet printing, the printed material is continuous rather than discrete droplets. The most important extrusion printing methods are the pneumatic (Fedorovich et al., 2009; Chang et al., 2011) or mechanical (piston or screw) (Visser et al., 2013) distribution systems. The setup with piston-driven deposition provides extended control overflow of the bioink, while screw-driven systems enable good spatial control and are useful for depositing highly viscous bioink. The pneumatic-driven system is helpful for depositing bioink of various types and viscosities by modulating the pressure and valve-gating time (Matai et al., 2020). Conversely, several studies have shown that there is a delay in the volume of compressed gas in the pneumatic system, while the mechanical distribution of flow material control is more precise in the screw-based system, and it can provide more space control and print hydrogels of higher viscosity (Chang et al., 2011). The hydrogels used in this type of printing generally belong to the category of non-Newtonian fluids, in which the viscosity is strongly dependent on the shear rate (Jungst et al., 2016). Typical viscosity values for this type of printing range from 30 to 6 × 10^7^ MPa/s (Chang et al., 2011; Holzl et al., 2016). The resolution of this method is generally 100 μm (Ozbolat and Yu, 2013; Derakhshanfar et al., 2018), but several studies have shown that its accuracy can reach 5 μm (Murphy and Atala, 2014).

The main advantage of extruded bioprinting is its ability to print very high cell density models (Bishop et al., 2017); high cell density scaffolds can provide more basic support for the recovery of spinal cord injury, which is favorable for spinal cord bioprinting. At the same time, continuous printing (line-by-line) can ensure that the distribution of cells in the scaffold is relatively average. However, the disadvantage of this technique is also clear—that is, a reduced cell survival rate. Studies have shown that the cell survival rate is significantly reduced with increasing bioink concentrations, an increase in viscosity leads to an increase in shear stress during extrusion (Yu et al., 2013). The way in which bioink is extruded also limits the printing resolution to hundreds of microns scale (He et al., 2016; Liu et al., 2017). By using this printing method the pressure affects the viability of cells, the nozzle diameter being one of the important parameters affecting its viability (Nair et al., 2009). Consequently, the optimization of printing equipment parameters is a key measure to improve the survival rate of cells. Recent studies have shown that cell survival rates of more than 90% after extruding bio-printed tissue can be obtained (Seol et al., 2014; Liu et al., 2021).

### 3.3 Light-Assisted Bioprinting

Light-assisted bioprinting included digital light processing (DLP), stereolithography (SLA), and laser-assisted bioprinting (LAB). It is a nozzle-less printing process, so its printing time is unaffected by the complexity of the printing structure. Moreover, this printing technique does not produce shear forces at the nozzle-head, so the printed tissue shows good cell viability and biocompatibility (Gauvin et al., 2012; Wang et al., 2015; Mandrycky et al., 2016; Derakhshanfar et al., 2018).

#### 3.3.1 Laser-Assisted Bioprinting

At present, LAB mainly originates from laser direct write (LDW) and laser-induced transfer technologies (LIFT) (Koch et al., 2012; Liao et al., 2012). LAB is mainly composed of three components a pulsed laser, a ribbon, and a receiving substrate. After the laser pulser emits the laser, the ribbon-containing metal material, such as gold (Au) or titanium (Ti), absorbs the laser, and then the biomaterials suspended at the bottom of the ribbon are evaporated by the laser pulse to form high-pressure bubbles, which are finally deposited on the receiving substrate to form the corresponding biological pattern (Keriquel et al., 2017; Schiele et al., 2010). (Figure 1C) LAB technology is not as common as extrusion and inkjet printing in the biological field, but it is also used in tissue engineering by some researchers. The typical viscosity values of bioink produced using this printing method are in the 1–300 MPa/s (Murphy and Atala, 2014; Jungst et al., 2016) range, and the scaffolds can be printed with an accuracy of up to 10 μm (Derakhshanfar et al., 2018). It can use a laser repetition rate of 5 kHz to deposit cells at a density of up to 10^8^ cells/mL and can achieve a printing resolution of just one cell per drop (Guillotin et al., 2010). Compared with the aforementioned advantages of LAB, it also has some disadvantages that cannot be ignored. First, faster gel dynamics are required to achieve high shape fidelity owing to the high resolution of the print, which results in longer print times and lower print flow (Guillotin and Guillemot, 2011). Second, multiple cell types and materials cannot be printed at the same time because of the limitations of the structure of such printing equipment, and this method may result in onerous and time-consuming workloads. Furthermore, some studies have shown the cell survival rate using this printing method to be below 85% (Hopp et al., 2012). This may be because of heat damage caused by the laser printing process. The problem of low cell viability caused by printer system is undoubtedly unfavorable to spinal cord bioprinting.

#### 3.3.2 Digital Light Processing

DLP bioprinting is based on the polymerization of light-sensitive polymers using precisely controlled light flashed from the digital micromirrors device (DMD) (Derakhshanfar et al., 2018). (Figure 1D) The manufacturing of 3D structure is completed by moving the working platform from bottom to up. UV laser is used to solidify the liquid, especially, covalent bonds between neighboring polymer chains are created by the energy supplied by the laser (Billiet et al., 2012). First, immerse the working platform in the liquid, and then excite the image on the DMD using the light source to form a layer of 2D image on the platform. Then, move the same amount of interlayer distance from bottom to up through the working platform to make the 3D structure stack layer-by-layer and finally form the target structure (Zhang et al., 2020). Compared to the serial printing process—that is, drop-by-drop or line-by-line printing—of the inkjet or extrusion printers, DLP projects an entire light pattern plane into a photopolymer solution, significantly increasing the time required for preparation. By continuously refreshing the projected optical patterns and moving the stage/printed object, smooth 3D objects can be printed with no artificial interfaces occurring between the droplets (as is the case with the inkjet printing) or lines (as is the case with the extrusion printing) (Zhang et al., 2012; Tumbleston et al., 2015; Zhu et al., 2016). When printing starts, this printing method can ensure printing accuracy and speed. Claire *et al.* and Yu et al. (2019) used DLP to print decellularized extracellular matrix (dECM)-based structures as fine as 30 μm, producing complex hierarchal branched geometries in mere seconds. This represented a significant improvement in printing resolution—that is, <100 μm—and an orders of magnitude with faster fabrication speed than that of extrusion-based systems. Kim et al. (2018) used DLP technology to print a hydrogel mixed with silk fibroin and alginate of low viscosity. The printing tissue overcame the problems related to nozzle stress caused by extrusion printing, achieving high printing accuracy with a resolution of 1 μm. Because the 3D structure manufactured in this way is to immerse the stage into a tank containing liquid for manufacturing layer-by-layer, rather than loading materials into the cartridge and the printing speed is very fast, this manufacturing process has very few setting effects, and because of the manufacturing method of layer-by-layer, it can more efficiently simulate the biological structure of the spinal cord. DLP technology is a relatively new technology, and hydrogels suitable for it remain to be explored.

#### 3.3.3 Stereolithography

Different from the DMD mode of DLP, SLA uses point or line scan laser crosslinking mode, and the rest is similar to DLP. Compared with other printing methods, this method has no limit on the cell viscosity (Mandrycky et al., 2016) and is also capable of printing tissue structures with a resolution of approximately 100 μm (Gauvin et al., 2012; Gou et al., 2014). Depending on the specific photo-initiator requirements, this printing method usually requires the use of ultraviolet or visible light to form covalent bonds in the bioink to achieve cross-linking without causing significant toxicity to the coated cells. Ultraviolet light is a common polymerization method, but studies have shown that it is harmful to DNA cells and may even lead to skin cancer (De Gruijl et al., 2001; Sinha and Hader, 2002). Consequently, in recent years researchers have focused their attention on visible light photopolymerization. Wang et al. (2015) used visible light as a cross-linking agent for their study, which was achieved by using a mixture of polyethylene glycol diacrylate (PEGDA) and GELMA hydrogels with an eosin Y-based photoinitiator. Their study showed that a method using visible light as a cross-linking agent could print a hydrogel with a resolution of 50 μm and an 85% cell survival rate. Sakai et al. (2018) also used visible light as a cross-linking agent, producing a phenyl-modified alginate saline gel with a cell damage rate (measured 7 days later) of approximately 5%. Although, SLA does not limit the precision of printing tissue and cell viscosity because of the limitations of its own mechanical structure, and printing principle processing times based on this method can be relatively slow, which cannot be ignored (Daly et al., 2021).

As mentioned earlier, inkjet printing faces great challenges in spinal cord bioprinting due to the limitation of its printing mode; extrusion printing is the most widely used and has been able to print scaffolds with high cell viability, which is undoubtedly beneficial to the spinal cord; DLP and SLA are relatively novel printing methods; however, due to their ability to produce scaffolds with high resolution, high cell viability, and highly complex geometrical features, these two technologies have become a potential alternative for spinal cord bioprinting.

## 4 Landmark Research of Spinal Cord Bioprinting

Due to the limitations of the traditional tissue engineering scaffold fabrication process, it cannot meet the requirements of complex structure manufacturing. Bioprinting can be divided into cell-laden and acellular ink printing. Compared with traditional tissue engineering scaffolds, acellular scaffolds made by bioprinting can have more meticulous spatial structure, and its “additive” manufacturing method can produce multi-dimensional linear structure, which conform to the spinal cord. Then, for cell-laden scaffolds, cells can be placed in a specific spatial position, and multi-cell scaffolds can also be made. This can meet the needs of the spinal cord for the direction of cell regeneration. With the increase of bioprinting, effectively manufacturing or recreating patient-specific constructs of clinically relevant size, shape, and structural integrity has been advanced using the combination of neural stem and progenitor cells with 3D printing biocompatible scaffolds to test new therapeutic options for SCIs (Joung et al., 2020). Table 2 summarizes the landmark research works of spinal cord bioprinting in recent years.

**TABLE 2:** Landmark research works of spinal cord bioprinting in recent years.

Cell type	Printing method	Bioink	Cross-link method	*In vitro*/*in vivo*	Nerve system type	Outcomes	References
NSCs	Extrusion	Collagen/heparin sulfate scaffold	UV light cross-link	*In vivo*	Central nerve system	Collagen/heparin sulfate scaffolds fabricated with a bioprinter could provide a permissive regeneration microenvironment by bridging the spinal cord lesion. The neuronal circuits were partially reestablished in rats with the collagen/heparin sulfate transplant	Chen et al. (2017)
NSCs	Extrusion	Collagen/silk fibroin scaffold	—	*In vivo*	Central nerve system	Collagen/silk scaffold shows good biocompatibility. Subsequent kinematics function tests also showed that the motor function of rats after implantation of scaffold was improved	Jiang et al. (2020)
NPCs	Microscale continuous projection printing method (μCPP)	PEGDA/GELMA	UV light cross-link	*In vivo*	Central nerve system	NPCs could differentiate into neurons and grow along the channels formed by the scaffold. Newborn neurons can grow along the scaffold channel and form a new “nerve relay."	Koffler et al. (2019)
NPCs/OPCs	Extrusion	AG/MC	Chemical cross-link	*In vitro*	Central nerve system	3D manufacture of neural tissue constructs in which different specific cell types can be precisely positioned within a neuro-compatible scaffold *via* a one-pot printing process	Joung et al. (2018)
EMSCs	Extrusion	SA-MA	Chemical cross-link	*In vitro*	Central nerve system	Printed scaffolds can promote the growth and proliferation of cells, and EMSCs can differentiate into neurons more effectively	Li et al. (2020)
NSCs	Extrusion	PEGDA/GELMA	UV light cross-link	*In vitro*	Central nerve system	Low-dose light could promote the differentiation of NSCs into neurons and inhibit the differentiation of glial cells	Zhu et al. (2017)
NSCs	Extrusion	HBC/HA -VS/HA-SH/MA	Chemical cross-link	*In vivo*	Central nerve system	The 3D bioprinted scaffold provides an ideal microenvironment for the growth and neural differentiation of NSCs, resulting in rapid and efficient restoration of locomotor function in the rat SCI model	Liu et al. (2021)

Chen et al. (2017) developed collagen/heparin sulfate scaffolds, which were used for extrusion bioprinting. In this study, a multi-dimensional grid scaffold with channels separated by approximately 400 μm was preapared, and the grid structure also had porous structure on the surface of the scaffold, which provided the structural basis of multi-dimensional distribution for subsequent cell inoculation. This structure could simulate the linear structure of the spinal cord, and the cells could grow relatively orderly along the channel of the scaffold after implantation. Considering that the mechanical properties of collagen are very soft relative to the spinal cord, the mechanical properties of collagen were improved by applying heparin sulfate modification to the scaffold; the results showed that the mechanical strength of the modified collagen was enhanced, and the strength of the modified scaffold after bioprinting was significantly enhanced. NSCs were then loaded onto biological scaffolds. Improvements in motor functions were observed after SCI. Jiang et al. (2020) also used collagen as a base and added silk fibroin protein to strengthen it to construct biological scaffolds. They also used extruded bioprinting technology and NSCs for inoculation. Different from Chen’s study, the scaffold model in this study attempted to imitate the butterfly-like structure of the gray matter of the spinal cord, which is generally an oval structure, with four linear pore structures in the middle gray of the matter and the surrounding white matter, and the rest is a solid structure. This structure is more consistent with the real structure of the spinal cord than that of the grid structure. The experimental results showed that NSCs adhered well and extended the scaffold, indicating that the scaffold had good biocompatibility. Subsequent kinematic function tests also showed that the motor function of rats improved after the implantation of the scaffold.

**FIGURE 2:**
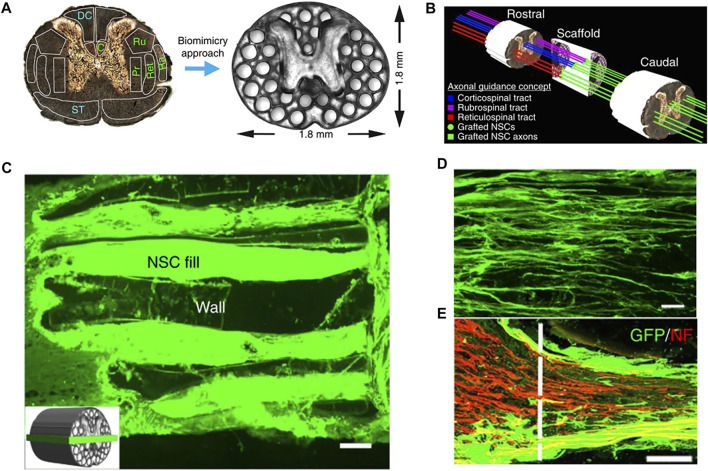
**(A)** PEGDA/GELMA hydrogel-based spinal cord scaffolds are printed, the gray matter is printed as a solid. The scaffold mimics the linear organization of white matter. Channels are precisely printed in 3D space. **(B)** Schematic diagram explaining the axonal alignment and guidance hypothesis. Channels in the scaffold provide linear guidance of rostral–caudal planes, so that grafted cells and host cells can be connected linearly. The host original axons regenerate in the scaffold and form synaptic connections with the neurons in the scaffold. The axons in the scaffold continue to extend to the lesion and form a functional connection at the caudal side of the host lesion. **(C)** Channels are filled with GFP-expressing NPCs. **(D)** Implanted GFP-expressing NSCs extend linear axons within the scaffold. Rostral is to the left and caudal is to the right. **(E)** Rostral entrance to the channel is penetrated by labeled NF host axons. Reproduced with permission from Koffler et al. (2019).

Koffler et al. (2019) developed a 3D-printed hydrogel spinal cord scaffold to evaluate spinal cord regeneration by planting cells on it (Figure 2). This study creatively developed a new microscale continuous projection printing technique (µCPP) using PEGDA–GELMA as an acellular hydrogel ink. This technology was improved based on DLP to achieve continuous printing. While inkjet or extrusion printing can compromise mechanical integrity through artificial interfaces between the printed drops or lines, µCPP’s layerless printed structures do not exhibit planar artifacts (interfaces). Similarly, this technology could print a biological scaffold suitable for the size of a patient’s spinal cord in 1.6 s and could also be used to customize the scaffold based on different shapes of human SCIs. They loaded neural progenitor cells (NPCs) onto bio-printed scaffolds in the hope that they would regenerate axons and thus restore functions after SCI. The experimental results showed that NPCs could be differentiated into neurons and grow along the channels formed by the scaffold. Cells were not only seen to enter the scaffold from the host end at the injured cranial side but regenerated axons were also seen to extend from the inside of the scaffold to the host end at the injured caudal side. They demonstrated that such “axon elongation” was capable of synaptic conduction and biological function. The results of their study are exciting and prove that 3D biomimetic scaffolds can be an effective option for promoting the regeneration of the spinal cord (Koffler et al., 2019).

As mentioned in the earlier studies, which pay more attention on the reconstruction of spinal cord structure, Zhu et al. (2017) focused on the study of cell differentiation. They applied GELMA and PEGDA as scaffold substrates and planted NSCs on biological scaffolds, and the channels of the scaffold were about 250 μm. Low-level light therapy (LLLT) has been shown to have positive effects on the rehabilitation of degenerative nerves and neural disorders. Consequently, low-dose light was used to explore the effects of light on the proliferation and differentiation of NSCs. The results showed that low-dose light promoted the differentiation of NSCs into neurons and inhibited the differentiation of glial cells. These findings suggested that integration of 3D printing and LLLT might provide a powerful methodology for neural tissue engineering (Zhu et al., 2017). Li et al. (2020) also focused on the differentiation of ectomesenchymal stem cells (EMSCs) but applied the cell-laden scaffold for research. They applied sodium alginate-Matrigel (SA-MA) hydrogel and extrusion bioprinting to reconstruct structures. At the same time, EMSCs were blended with hydrogel to form bioink as cell-laden. Traditional 2D cell culture was used for the control group, in which the cells were cultured directly in the culture medium, focusing on evaluating the differentiation of EMSCs between cell-laden and 2D culture. Experimental results showed that compared with traditional 2D cell culture, the microenvironment provided by 3D-printed scaffolds could promote the growth and proliferation of cells, and EMSCs could differentiate into neurons more effectively. The results of their study provided a new strategy for the differentiation of cells and the application of biological scaffolds in the treatment of SCIs.

Joung et al. (2018) also developed a cell-laden scaffold based on extrusion technology, using alginate (AG) and methylcellulose (MC) for printing. In contrast to other studies, this study enabled multiple neural cell types to be co-printed in a specific channel—clusters of NPCs and oligodendrocyte progenitor cells (OPCs) were placed with a spatial distribution of 200 µm within 150 µm wide channels. They expected the cells could differentiate into neurons and oligodendrocytes, then formed myelinated nerve fibers, and finally, while exploring the directional differentiation of cells, they could accurately locate cells in space. The experimental results showed that both types of progenitor cells could grow along the scaffold channel and extend and differentiate into corresponding cell lines, these results provided an important experimental basis for cell regeneration in spinal cord tissue engineering. It was also confirmed that AG combined with MC was the only material capable of carrying a variety of neural precursor cell types in the process of 3D bioprinting. In addition to differentiating NSCs into neurons, they could also differentiate into other neuron-related tissues. To repair the biological function of a damaged nerve, it is necessary to ensure the NSCs differentiate in the direction of the neuron as only in this way synapses structures can be formed and biological functions can be generated. This study proposed a new biological scaffold, which provided new ideas for simulating the structure of the nervous system *in vitro* and provided an option for the future development of new clinical treatment methods for SCIs.

**FIGURE 3:**
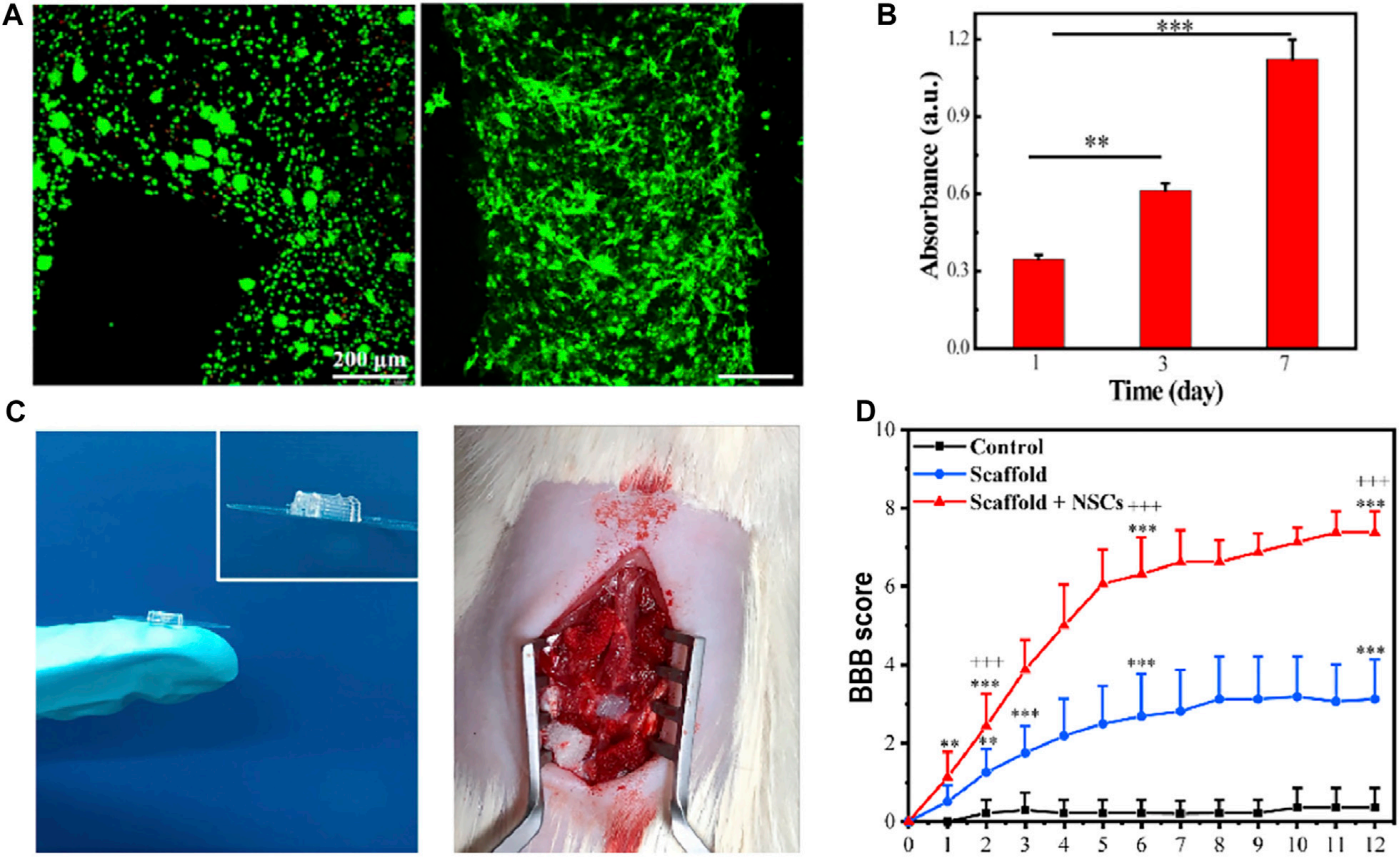
**(A)** Live/dead staining of NSCs within the 3D bioprinted NSC-laden HBC/HA/MA scaffold cultured for 0 days (left) and 7 days (right), respectively. High cell viability can be observed. **(B)** Proliferation of NSCs in the 3D bioprinted scaffold after culture for 1, 3, and 7 days **(C)** General diagram of the 3D bioprinted scaffold and the implantation of the scaffold into the gap of SCI lesion. **(D)** BBB score after the implantation of the scaffold for different weeks. Reproduced with permission from Liu et al. (2021).

Liu et al. (2021) developed a composite hydrogel scaffold composed of hydroxypropyl chitosan (HBC), thiolated hyaluronic acid (HA-SH), vinyl sulfonated hyaluronic acid (HA-VS), and Matrigel (MA). This study is the first *in vivo* experimental validation study using the cell-laden scaffold; the scaffold was also a multi-dimensional grid structure with 400 μm channels. When printed, the cells were evenly distributed in the grid hydrogel. They used extrusion printing, the cell survival rate being approximately 95%. The results of *in vitro* experiments showed that the composite scaffold had a degradation rate, porosity, and mechanical strength suitable for the growth of NSCs, providing an ideal microenvironment for the proliferation and differentiation of NSCs. The results of *in vivo* experiments and biomechanical measurements showed that the scaffold simulated the parallel linear structure of the spinal cord, realized the regeneration and connection of neurons, and promoted the recovery of motor functions in SCI models. The results of their study have promoted the development of the cell-laden scaffold in the regeneration of spinal cord injury (Figure 3).

## 5 Summary, Challenges, and Outlook for Spinal Cord Bioprinting

Bioprinting is emerging as a promising tool in tissue engineering, providing bioengineering researchers with the ability to design complex 3D biological structures. It can print specific nerve cell subtypes and growth factors in a region-specific manner, promoting the differentiation of NSCs into neurons using the ratio of different components of bioink, providing the precisely orchestrated re-establishment of neural networks and connections. This provides a new direction for the repair of SCIs. Bioprinting technology can also be used to create individualized SCI models to test the feasibility of a treatment plan, including the biocompatibility of the cells and scaffolds, cell–cell and cell–matrix interactions with cell–matrix interactions, and even improve our understanding of the mechanisms of neuromodulation—in short, 3D printing can provide personalized treatment for SCIs. It can create unique models for different individuals with different injuries and is more in line with the “from the bottom up” regeneration of the body compared to traditional scaffolds.

However, although bioprinting has many advantages, it still faces many challenges. To date, only a few specific cell types and scaffold models have been studied in spinal cord bioprinting. As mentioned earlier, researchers pay attention to the differentiation of NSCs and the production of scaffold three-dimensional structure, but the structure of scaffold only focuses on the production of multi-dimensional grid structure. Although this simulates the structure of spinal cord linear conduction bundle, it does not completely restore the butterfly-like gray matter and surrounding white matter structure of the spinal cord. Current bioprinting technology has been able to simulate a variety of organs in the body, but the spinal cord has a complex tissue structure and many corresponding neural pathways, making it extremely challenging to complete bionic reconstruction.

At present, bioprinting for the spinal cord has not been combined with relevant studies such as vascularization, immunosuppression, and inflammation; this gives it a lot of room for development. To this end, it is necessary to encourage interdisciplinary research in multiple disciplines to promote the techniques which may have the greatest impact on SCI repair and provide guidance for the transformation of basic experiments into clinical efficacy. The cell-laden scaffold has been successfully used in the *in vivo* experiment of spinal cord injury, it is reasonable to believe that with the development of bioprinting technology, it will not be difficult to make a scaffold that fits the spinal cord structure more closely.
